# Identification of independent factors affecting bone mineral density after successful parathyroidectomy for symptomatic hyperparathyroidism

**DOI:** 10.1186/s12902-020-00622-4

**Published:** 2020-09-14

**Authors:** Shuai Lu, Maoqi Gong, Yejun Zha, Aimin Cui, Chen Chen, Weitong Sun, Kehan Hua, Wei Tian, Xieyuan Jiang

**Affiliations:** 1 Department of Orthopedic Trauma, Peking University Fourth School of Clinical Medicine, Beijing Jishuitan Hospital, Beijing, 100035 China; 2 Department of General Surgery, Peking University Fourth School of Clinical Medicine, Beijing Jishuitan Hospital, Beijing, 100035 China; 3 Department of Spine Surgery, Peking University Fourth School of Clinical Medicine, Beijing Jishuitan Hospital, Beijing, 100035 China

**Keywords:** Bone mineral density, Parathyroidectomy, Severe hyperparathyroidism

## Abstract

**Background:**

Studies have shown that the response of bone mineral density (BMD) to parathyroidectomy for symptomatic primary hyperparathyroidism (PHPT) is heterogeneous and difficult to predict. However, the independent factors affecting BMD in PHPT patients after parathyroidectomy remains limited and inconclusive. This study aimed to explore the independent factors affecting BMD changes in symptomatic PHPT patients after parathyroidectomy.

**Methods:**

This study retrospectively analyzed 105 patients with symptomatic PHPT treated at Beijing Jishuitan Hospital between January 2010 and December 2015. The primary outcome was a > 10% increase in BMD at 3 years after parathyroidectomy compared with the preoperative value, whereas the secondary outcomes were BMD changes at various measurement sites.

**Results:**

A total of 105 patients with a mean age of 46.37 years were included in this study. Univariate logistic regression analysis indicated that hypertension (odds ratio [OR[: 0.032; 95% confidence interval [CI]: 0.001–0.475; *P* = 0.012), and parathyroid hormone level (OR: 1.006; 95% CI: 1.004–1.009; *P* = 0.044) were associated with the > 10% BMD increase. However, these results were not significant after adjustments for potential confounders. Moreover, the BMD values at the lumbar spine, femoral neck, femoral trochanter, Ward’s triangle, and whole body after parathyroidectomy were significantly greater than those before the operation (*P* < 0.05).

**Conclusions:**

This study suggests that patient characteristics were not associated with the > 10% BMD increase. However, the BMD values of the femur and lumbar spine were significantly increased in symptomatic PHPT patients after parathyroidectomy.

## Background

Primary hyperparathyroidism (PHPT) is the third most common endocrine disorder, with a prevalence ranging from 0.1 to 0.7% in adults [1–3]. It remains a common cause of hypercalcemia and predominantly affects the elderly, especially women [4]. Although the disease is frequently associated with bone problems, most PHPT patients are asymptomatic [5]. Previous studies have demonstrated that bone mineral density (BMD), assessed using dual-energy x-ray absorptiometry (DXA), is significantly lower in patients with PHPT than in healthy individuals [6, 7]. However, whether the characteristics of patients could affect the changes in BMD after parathyroidectomy for PHPT is still not fully understood [8].

PHPT is significantly associated with hypercalcemia and high parathyroid hormone (PTH) levels, which could promote calcium reabsorption in the distal renal tubules and alter the conversion of 25(OH)-vitamin D to 1,25(OH)2-vitamin D through 1α-hydroxylase stimulation [9]. Moreover, PTH has anabolic effects on trabecular sites and catabolic effects on cortical sites, which depend on the secretion levels and the duration of exposure to high PTH levels. Studies have reported an increased risk of fractures at various sites in patients with PHPT, and this risk was significantly reduced after parathyroidectomy [10, 11]. Furthermore, the changes in BMD in patients treated conservatively or by parathyroidectomy were found to be significant [12, 13]. This study aimed to explore the potential effect of patients’ characteristics on BMD values before and after parathyroidectomy in patients with symptomatic PHPT. Moreover, the changes in BMD at various sites before and after parathyroidectomy in patients with symptomatic PHPT were also compared.

## Methods

### Study design

In this retrospective study, we evaluated the preoperative and postoperative BMD values in patients with symptomatic PHPT who underwent successful parathyroidectomy. This study was approved by the Ethics Committee of Beijing Jishuitan Hospital and informed consent was not required owing to the retrospective design of the study.

### Patients

A total of 105 patients with symptomatic PHPT at Beijing Jishuitan Hospital, China, were recruited between January 2010 and December 2015. All patients underwent successful parathyroidectomy and were followed up from the time of diagnosis up to 36.0 months postoperatively. The diagnosis of PHPT was made mainly according to high or inappropriate PTH levels and the presence of hypercalcemia. Patients were included if they met the following criteria: (1) serum PTH level > 65 pg/mL and serum calcium level > 2.75 mmol/L; (2) parathyroid lesion excision performed by experienced physicians in the same department; (3) biochemical and BMD measurement before and after parathyroidectomy; and (4) patients diagnosed with symptomatic PHPT. Patients were excluded if they met the following criteria: (1) incomplete BMD measurements before and after parathyroidectomy or patients who could not be followed up; (2) normal parathyroid gland tissue (i.e. no hyperplasia, adenoma, and parathyroid cancer) diagnosed by histopathological examination after excision of the parathyroid lesions; and (3) serum calcium level remained above the normal range after excision of the parathyroid lesions.

### Clinical characteristics

After admission, all patients underwent general physical examination and systemic evaluation for chronic diseases, performed by specially trained physicians using a pre-structured questionnaire. The collected information included sex, age, height, weight, body mass index (BMI, kg/m^2^), history of smoking, alcohol consumption, cardiovascular disease, bone pain, fracture, urinary calculus, and hypertension as well as parathyroid nature, wet weight, and volume. The urinary calculus status included the specific location of the calculi in the urinary system and a surgical history of urinary calculus. The clinical manifestations in the skeletal system included the location of malformation, degree of bone pain, location and timing of previous fractures, and weakness. The parathyroid nature, wet weight, and volume were measured by a professional pathologist.

### Biochemical and BMD measurements

Blood biochemical examinations were performed upon diagnosis and at 1 day after parathyroidectomy and subsequently for 36 months after parathyroidectomy. PTH was measured using an immunoelectrochemical analyzer (E601; Roche Diagnostics, Basel, Switzerland; normal range 15–65 pg/mL, intra-assay variation 2.38%, interassay variations 6.7 and 7.5%). Serum calcium, phosphorus, and alkaline phosphatase (ALP) levels were measured using standard automatic assays. The BMD values before and at 36 months after parathyroidectomy were measured using DXA with a lunar DPX (GE Healthcare, USA) in the array (fan beam) mode at the lumbar spine (L1–L4), total hip, femoral neck, femoral trochanter, and Ward’s triangle.

### Outcome measurements

The primary outcome was a > 10% increase in BMD at 3 years after parathyroidectomy when compared to the preoperative BMD value. The secondary outcomes included the BMD changes in the lumbar spine (L1–L4), total hip (femoral neck, femoral trochanter, and Ward’s triangle), and whole body after parathyroidectomy.

### Statistical analysis

Continuous and categorical data of the patient characteristics are presented as means and standard deviations and as event rates, respectively. Comparisons between continuous variables before and after parathyroidectomy were performed using paired *t* tests. Both univariate and multivariate logistic regression analyses were performed to explore the role of patient characteristics in the improvement of BMD. All reported *P*-values are two-sided and *P*-values < 0.05 were considered to indicate statistical significance. Data were analyzed using IBM SPSS Statistics for Windows, version 19.0 (SPSS 19.0).

## Results

### Baseline characteristics

The mean age of the patients was 46.37 ± 16.61 years, and 62% were women (32% of the women were in the menopausal state). The baseline characteristics of the recruited patients are summarized in Table 1. All patients were diagnosed with symptomatic PHPT accompanied by at least one of the following conditions: bone pain, fracture within the last 10 years, urinary calculus, cardiovascular disease, and hypertension. Bone pain was present in up to 67% of the patients, and a history of fracture within the last 10 years was noted in 35%; these were the first presenting symptoms in most patients. Postoperative pathological results revealed that the incidence of parathyroid adenoma was up to 90%.

**Table 1:** The baseline characteristics of enrolled patients

Variable	Results
Age (years)	46.37 ± 16.61
Male (%)	38
Female (%)	62
Menopause (%)	32
*BMI (kg/m^2^)	23.03 ± 3.51
Smoking (%)	17
Alcohol (%)	5
*CVD (%)	13
Bone pain (%)	67
Fracture^a^ (%)	35
Urinary calculus (%)	34
Hypertension (%)	31
*PTH (pg/mL)	1053.44 ± 1068.37
Calcium (mmol/L)	2.89 ± 0.35
Phosphorus (mmol/L)	0.72 ± 0.22
*ALP (U/L)	554.32 ± 663.85
Parathyroid nature	
Hyperplasia (%)	5
Adenoma (%)	90
Cancer (%)	5
Wet weight (gram)	5.32 ± 7.72
Volume (cm^3^)	10.37 ± 13.84

^a^:fracture occurred within 10 years; *ALP alkaline phosphatase, *BMI* body mass index, *CVD* cardiovascular disease, *PTH* parathyroid hormone

### Primary outcome

Univariate logistic regression analysis indicated that hypertension (*P* = 0.012) and PTH level (*P* = 0.044) were associated with postoperative BMD improvement, while sex (*P* = 0.792), age (*P* = 0.901), BMI (*P* = 0.533), cardiovascular disease (*P* = 0.602), bone pain (*P* = 0.701), fracture (*P* = 0.263), urinary calculus (*P* = 0.122), calcium level (*P* = 0.700), serum phosphorus level (*P* = 0.062), ALP level (*P* = 0.136), parathyroid nature (*P* = 0.654), wet weight (*P* = 0.097), and volume of the removed parathyroid gland (*P* = 0.462) were not associated with postoperative BMD improvement (Table 2). However, multivariate logistic regression analysis demonstrated that hypertension (*P* = 0.229), PTH level (*P* = 0.171), serum phosphorus level (*P* = 0.215), and wet weight of the removed parathyroid gland (*P* = 0.259) were not associated with postoperative BMD improvement (Table 2).

**Table 2:** Univariate and multivariate model analysis of predictors of postoperative bone mineral density improvement (BMD > 10%)

Parameter	n	OR	95% CI		*P* value
			Lower	Upper	
**Univariate logistic models**	N				
Sex	105	1.291	0.201	8.432	0.792
Age	105	1.012	0.933	1.084	0.901
*BMI	96	0.922	0.662	1.245	0.533
*CVD	14	1.678	0.154	18.871	0.602
Bone pain	70	0.813	0.273	2.454	0.701
Fracture^a^	37	3.891	0.373	41.352	0.263
Urinary calculus	36	6.432	0.614	68.313	0.122
Hypertension	32	0.032	0.001	0.475	**0.012**
*PTH	103	1.006	1.004	1.009	**0.044**
Calcium	103	1.882	0.076	46.793	0.700
Phosphorus	102	0.003	0.000	1.352	0.062
*ALP	101	1.002	0.999	1.005	0.136
Parathyroid nature	100	1.861	0.173	16.638	0.654
Wet weight	100	1.494	0.930	2.400	0.097
Volume	98	1.046	0.929	1.177	0.462
**Multivariate logistic models**					
Hypertension	32	0.161	0.008	3.156	0.229
*PTH	103	1.003	0.999	1.007	0.171
Phosphorus	102	0.001	0.000	51.379	0.215
Wet weight	100	2.299	0.542	9.742	0.259

^a^:fracture occurred within 10 years; *ALP alkaline phosphatase, *BMI* body mass index, *CVD* cardiovascular disease, *PTH* parathyroid hormone

### Secondary outcomes

**Fig. 1:**
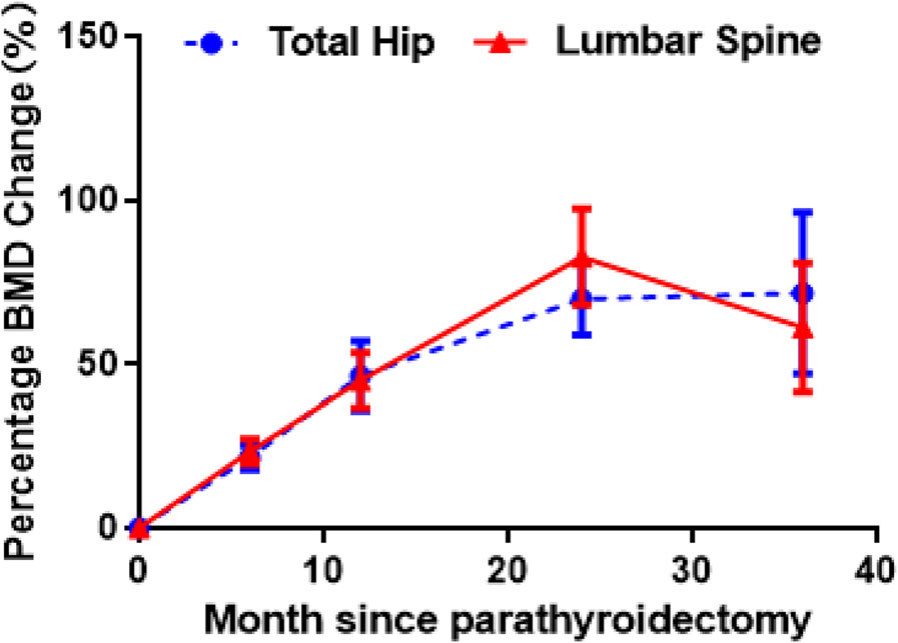
The changes of BMD in lumbar spine and total hip after parathyroidectomy

**Fig. 2:**
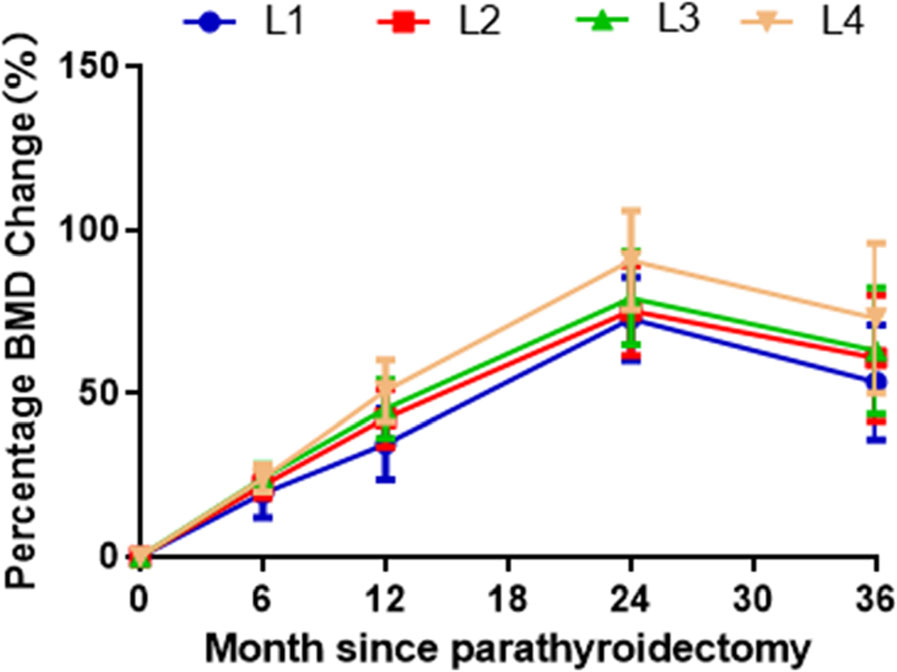
The changes of BMI in specific sites of lumbar spine after parathyroidectomy

**Fig. 3:**
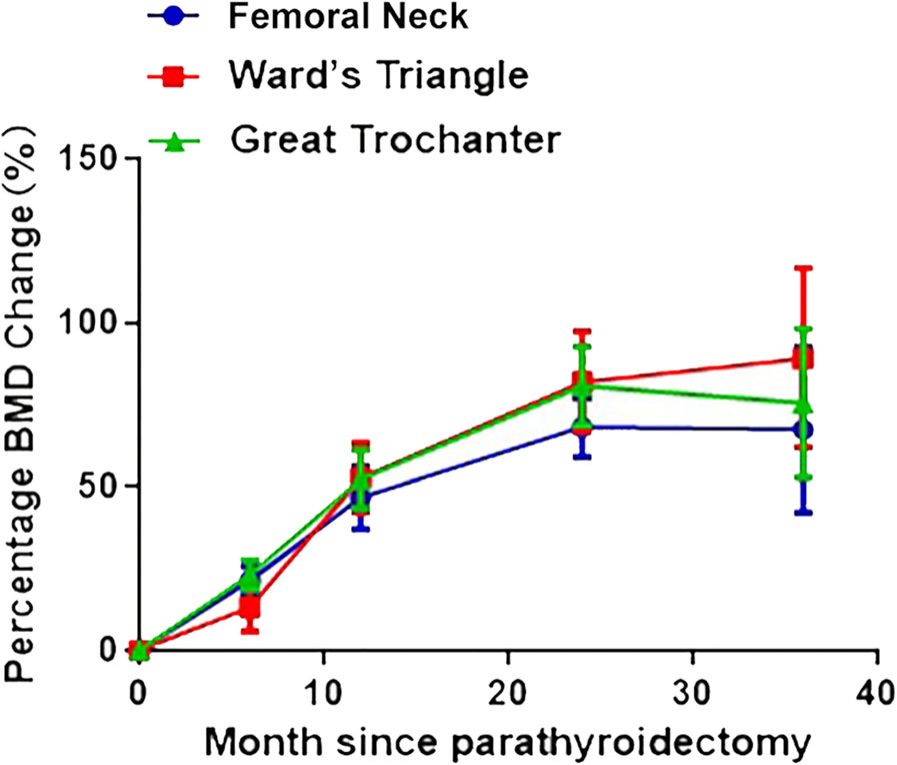
The changes of BMI in femoral neck, Ward’s triangle, and great trochanter after parathyroidectomy

The BMD measurements before and at 12.0 months after parathyroidectomy in the lumbar spine (L1–L4), total hip (femoral neck, femoral trochanter, and Ward’s triangle), and whole body are shown in Table 3. The BMD values after parathyroidectomy were significantly higher than the preoperative values in all the measurement sites (*P* < 0.05). The BMD changes from the preoperative values to the follow-up measurements at 6, 12, 24, and 36 months in the lumbar spine and total hip are presented in Fig. 1. The BMD values in the lumbar spine and total hip were significantly increased compared with the preoperative values, and the rate of BMD increase after 36 months of follow-up showed a gradual decrease compared with that in the previous 2 years. Furthermore, the BMD values of all parts of the lumbar spine were significantly increased compared with the values before parathyroidectomy, and the increase in BMD after 36 months of follow-up was slower than that in the previous 2 years. In the lumbar spine, the increase in BMD in L1 was the least, while that in L4 was the most evident (Fig. 2). Finally, the BMD values of all parts of the total hip showed a significant increase compared with the preoperative values, whereas the BMD increase after 36 months gradually slowed down. The BMD increase in the femoral neck was the least, whereas the BMD improvement in the Ward’s triangle was the highest (Fig. 3).

**Table 3:** The changes of BMD before and after parathyroidectomy

Variable	Preoperative (g/cm^2^)	12.0 months after parathyroidectomy (g/cm^2^)	*P* value	95% CI
lumbar spine				
L1	0.77 ± 0.19	1.07 ± 1.18	0.000	−0.40—-0.19
L2	0.81 ± 0.22	1.10 ± 0.21	0.000	−0.41—-0.17
L3	0.85 ± 0.23	1.18 ± 0.24	0.000	−0.46—-0.21
L4	0.84 ± 0.24	1.17 ± 0.24	0.000	−0.46—-0.20
L1-L4	0.82 ± 0.22	1.13 ± 0.21	0.000	−0.43—-0.20
Total hip				
Femoral neck	0.64 ± 0.16	0.90 ± 0.20	0.000	−0.35—-1.6
Great trochanter	0.52 ± 0.17	0.71 ± 0.17	0.000	−0.28—-0.97
Ward’s triangle	0.46 ± 0.16	0.67 ± 0.25	0.006	−0.34—-0.07
All	0.65 ± 0.20	0.88 ± 0.20	0.000	−0.34—-0.12

## Discussion

This study provides comprehensive results pertaining to the changes in BMD at various sites and demonstrates the potential effect of patient characteristics over a period of 3 years after successful parathyroidectomy in patients with symptomatic PHPT. This quantitative retrospective study included 105 symptomatic PHPT patients witha wide range of characteristics. Although the crude results suggested that hypertension and PTH level were significantly associated with BMD improvement, these results were not significant after adjustments for potential confounders. Moreover, the BMD values of all parts of the lumbar spine, total hip, and whole body after parathyroidectomy were significantly higher than the preoperative values. Furthermore, an increase in the BMD values in all sites after parathyroidectomy was obvious within the first 2 years, and this increasing trend gradually slowed down after 3 years of follow-up. Finally, the improvement in the BMD at L4 in the lumbar spine and the Ward’s triangle was the highest compared to that in other parts.

Studies have already reported changes in BMD after parathyroidectomy in patients with symptomatic PHPT and found that bone damage is reversible after successful parathyroidectomy [14, 15]. Nakaoka et al. discovered that the BMD significantly increased in patients after parathyroidectomy, even in asymptomatic patients without a surgical indication. They reported that the BMD values of the lumbar spine and forearm increased by 12.2 and 11.6%, respectively [14]. Moreover, Silverberg et al. reported that the BMD increased at the lumbar spine and femoral neck by 8.2 and 5.9%, respectively, and the improvement in BMD after the third year following parathyroidectomy was 4% [15]. Hesp et al. claimed that the BMD increase in the cortical sites in the first year after parathyroidectomy was not significant, whereas the increase seen at 2 years after parathyroidectomy was significant in patients with parathyroid adenoma [16]. This difference may be explained by the fact that the metabolic rates in the trabecular and cortical bone were 30 and 3%, respectively, and the higher bone turnover in the trabecular sites could be attributed to the rapid BMD improvement after parathyroidectomy compared to that in the cortical sites [17].

The results of this study suggest the potential association of hypertension and PTH level with postoperative BMD improvement. However, these results were not significant after adjusting for potential confounders. Consequently, it can be considered that patient characteristics did not have a significant effect on postoperative BMD improvement. Ohe et al. recruited 31 patients with PHPT and found a significant association between the preoperative level of turnover markers and improvement in BMD 1 year after parathyroidectomy [18]. Therefore, these results suggest that the preoperative levels of turnover markers could be predictors of BMD improvement after parathyroidectomy. Moreover, a study conducted by Lee et al. indicated that the skeletal benefit of parathyroidectomy was attenuated in normocalcemic and normohormonal patients, which might be attributed to the fact that the skeletal changes occurring after parathyroidectomy mainly depend on the biochemical profile [19]. A prospective database study conducted by Sharma et al. reported that sex, age, severe preoperative bone disease, and insurance status were significantly associated with greater BMD improvement after parathyroidectomy [20]. Nevertheless, these findings were not observed in the current retrospective study, which may be attributable to the use of > 10% BMD improvement as the study endpoint.

Several limitations of this study should be mentioned: (i) since this study was designed as a retrospective analysis, potential uncontrolled biases were inevitable; (ii) the use of background therapies was not addressed, and these therapies might have played an important role in BMD changes after parathyroidectomy; (iii) stratified analyses based on the patients’ characteristics were not conducted because of the small sample size and the wide range of 95% confidence intervals; and (iv) the severity of PHPT, which could affect the improvement in BMD, was not quantitatively assessed.

## Conclusion

The results of this study indicate that patients’ characteristics were not associated with postoperative BMD improvement. Moreover, the improvement in BMD after parathyroidectomy in all measurement sites was significant. Furthermore, the increase in BMD was evident within the first 2 years after parathyroidectomy, and this trend subsequently slowed down after 3 years of follow-up. Further large-scale prospective cohort studies are required to identify potential predictors for the improvement in BMD in symptomatic PHPT patients.

## Data Availability

The datasets used and/or analyzed during the current study are available from the corresponding author on reasonable request.

## Abbreviations

BMD: Bone mineral density
DXA: Dual-energy x-ray absorptiometry
PHPT: Primary hyperparathyroidism
PTH: Parathyroid hormone
